# Acute and Chronic Performance Enhancement in Rowing: A Network Meta-analytical Approach on the Effects of Nutrition and Training

**DOI:** 10.1007/s40279-023-01827-y

**Published:** 2023-04-25

**Authors:** Steffen Held, Ludwig Rappelt, Lars Donath

**Affiliations:** 1 Department of Intervention Research in Exercise Training, Institute of Exercise Training and Sport Informatics, German Sport University, Cologne, Germany; 2 Department of Sport and Management, IST University of Applied Sciences, Duesseldorf, Germany; 3 Department of Movement and Training Science, University of Wuppertal, Wuppertal, Germany

## Abstract

**Introduction:**

This systematic review and network meta-analysis assessed via direct and indirect comparison the occurrence and magnitude of effects following different nutritional supplementation strategies and exercise interventions on acute and chronic rowing performance and its surrogates.

**Methods:**

PubMed, Web of Science, PsycNET and SPORTDiscus searches were conducted until March 2022 to identify studies  that met the following inclusion criteria: (a) controlled trials, (b) rowing performance and its surrogate parameters as outcomes, and (c) peer-reviewed and published in English. Frequentist network meta-analytical approaches were calculated based on standardized mean differences (SMD) using random effects models.

**Results:**

71 studies with 1229 healthy rowers (aged 21.5 ± 3.0 years) were included and two main networks (acute and chronic) with each two subnetworks for nutrition and exercise have been created. Both networks revealed low heterogeneity and non-significant inconsistency (*I*^2^ ≤ 35.0% and Q statistics: *p* ≥ 0.12). Based on P-score rankings, while caffeine (P-score 84%; SMD 0.43) revealed relevantly favorable effects in terms of acute rowing performance enhancement, whilst prior weight reduction (P-score 10%; SMD − 0.48) and extensive preload (P-score 18%; SMD − 0.34) impaired acute rowing performance. Chronic blood flow restriction training (P-score 96%; SMD 1.26) and the combination of β-hydroxy-β-methylbutyrate and creatine (P-score 91%; SMD 1.04) induced remarkably large positive effects, while chronic spirulina (P-score 7%; SMD − 1.05) and black currant (P-score 9%; SMD − 0.88) supplementation revealed impairment effects.

**Conclusion:**

Homogeneous and consistent findings from numerous studies indicate that the choice of nutritional supplementation strategy and exercise training regimen are vital for acute and chronic performance enhancement in rowing.

## Key Points

**Table:** 

While caffeine supplementation increases acute rowing time-trial performance, prior weight reduction or extensive preload could impair performance.
Chronic rowing time-trial performance was increased via blood flow restriction training and the combination of β-hydroxy-β-methylbutyrate and creatine supplementation.
In contrast, chronic spirulina and black currant supplementations might impair chronic rowing performance adaptations.

## Introduction

Rowing is considered a strength [1] endurance sport [2] that has been part of the Olympic program since 1896 [3]. In addition to a high and primarily aerobic endurance capacity [4], strength capabilities are crucial in rowing [1]. Therefore, 2000-m time trials are considered the gold standard for rowing performance testing [5, 6].

Rowing performance improvements were elicited via numerous different nutritional and exercise-based approaches, such as (i) resistance training [7], (ii) plyometric training [8, 9], (iii) breathing against resistance as respiratory training [10, 11], (iv) sprint interval training [12, 13], (v) high-intensity training [14, 15], (vi) blood flow restriction methods [16], (vii) altitude training [17, 18], and (viii) various nutritional supplementation strategies [19].

Furthermore, the enhancement of acute 2000-m timetrial performance was intended via (i) postactivation potentiation [20], (ii) respiratory preconditioning [21], (iii) precooling [22], (iv) weight loss management [23], or (v) nutritional supplementation [19]. In the context of nutritional supplementation, β-alanine [24], spirulina [25], black currant [26], elk velvet antler [27], creatine monohydrate [28], beetroot [29], sodium bicarbonate [30], and sodium citrate [31] were used. Despite the multitude of different acute and chronic interventional approaches, only few rowing-specific meta-analyses on nutritional supplementation strategies [19] and exercise interventions [7, 32] are available. Thereby, the effects of resistance training [7], preconditioning [32], and caffeine [19] have been meta-analytically reviewed only via direct pairwise comparisons. Accordingly, the rowing-specific findings on plyometric training [8, 9], respiratory training [10, 11], sprint interval training [12, 13], high-intensity training [14, 15], blood flow restriction methods [16], altitude training [17, 18], weight loss management [23], β-alanine [24], spirulina [25], black currant [26], elk velvet antler [27], creatine monohydrate [28], beetroot [29], sodium bicarbonate [30], and sodium citrate [31] have not yet been examined via meta-analytical approaches. This is partly explained by the lack of a sufficient number of studies to perform pairwise meta-analyses in each case. Therefore, the evidence resulting from pairwise comparisons does not sufficiently provide compelling evidence and does not allow for well informed decision-making by trainers, athletes, and practitioners in the field of rowing-related training, preconditioning, and nutritional strategies. Hence, a network meta-analysis (NMA) rather than pairwise approaches can address this issue adequately by accounting for direct and indirect comparisons of different interventions [33]. A NMA does not require experimental studies to include similiar comparators, the evidence that can be integrated for the relative comparison of different intervention types is extended and more comprehensive [33]. Since a NMA enables the comparison of numerous different intervention and treatment approaches, all the above-mentioned rowing-specific findings could be examined within one analysis. In addition, a NMA approach enables a treatment ranking based on effectiveness [34].

Against this background, the present systematic review and NMA aimed to examine and compare the effects of different nutritional and exercise-based interventions on acute and chronic rowing performance through indirect and direct network-analytical comparisons. The overall results might enable athletes and coaches to select evidence-based strategies to improve rowing performance acutely and chronically, respectively.

## Methods

### Search and Screening Procedures

This network-analytical review was conducted in accordance with Preferred Reporting Items for Systematic Reviews and Meta-Analyses for Network Meta-Analyses (PRISMA-NMA) (Hutton et al. 2015). The literature search and screening processes were independently conducted by two researchers (LR and SH). Four health-related, biomedical, and psychological databases (PubMed, Web of Science, PsycNET, and SPORTDiscus) were screened from inception until March 7, 2022. Relevant search terms (operators) were combined with Boolean conjunctions (OR/AND) and applied to three search levels (Table 1). In addition, tracking of cited articles and manual searching of relevant primary articles and reviews were also carried out. Duplicates were removed and the remaining studies underwent manual screening. The remaining studies were gradually screened using (i) titles, (ii) abstracts, and (iii) full texts for potentially eligible articles. Two researchers (LR and SH)made the final decision regarding inclusion or exclusion. The following inclusion criteria were applied based on the PICOS approach [population (P), intervention (I), comparators (C), main outcome (O), and study design (S)] : Full-text article published in English in a peer-reviewed journal; participants were healthy rowers (P), without any cognitive, neurological, orthopedic, and/or cardiac conditions that could affect physical testing and training; acute (≤ 7 days) or chronic (> 7 days) treatments or interventions (I); active and/or passive inactive control group(s) that received a placebo treatment or did not receive any intervention served as a comparator (C); at least one rowing-related outcome such as time trial (≥ 500 m), time to exhaustion, maximal oxygen consumption (*V*O_2max_), power at *V*O_2max_, or power at given lactate concentration (O); and prospective two- or multi-armed controlled intervention study with pre- and post-testing (S). The exclusion criterion was an inadequate control condition, which made integration into the network impossible.

**Table 1:** Search strategy

Search level	Search terms with Boolean operators
Search #1	“rowing” OR “rower” OR “row” OR “oarsmen”
Search #2	#1 AND (“VO2peak” OR “*V*O_2max_” OR “maximal oxygen uptake” OR “maximal oxygen consumption” OR “aerobic capacity” OR “threshold” OR “time trial” OR “time to exhaustion” OR “one repetition maximum” OR “1RM” OR “1 repetition maximum” OR “MVC” OR “maximal voluntary contraction” OR “rowing performance”)
Search #3	#2 NOT (“patient” OR “patients”)

### Assessment of Methodological Quality of the Studies

The methodological quality (including risk of bias) of the included studies was independently rated by two researchers (LR and SH) using the PEDro (Physiotherapy Evidence Database) scale [35]. The PEDro scale consists of 11 dichotomous (yes or no) items, in which criteria 2–9 rate randomization and internal validity, and criteria 10–11 rate the presence of statistically replicable results. Criterion 1 merely relates to external validity and was not  includeed PEDro score sum. Studies with a PEDro score ≥ 6 on a scale of 0 to 10 [35] we considered high-quality study.

### Data Extraction

Relevant data (required for calculating effect sizes) were extracted independently by two researchers ( LR and SH) using a standardized extraction Excel spreadsheet adapted from the Cochrane Collaboration [36]. Means and standard deviations of pre- and post-test scores on rowing-related performance outcomes were extracted along with the number of participants assessed in each group. If these point and variability measures were not reported in the full-text article, either the means and pooled within-group standard deviations of change scores were entered in an electronic spreadsheet or the authors were contacted and missing values were requested up to three times. If studies only presented means and standard deviations in figures, WebPlotDigitizer Version 4 (Free Software Foundation, Boston, MA, USA) was used to extract means with standard deviations [37]. WebPlotDigitizer was used in 10 studies. Data from three author requests are included. For acute effects, only time-trial performance was extracted. The following ranking was used to select respective outcome parameters for chronic effects: time trial > time to exhaustion > power at *V*O_2max_ > power at a blood lactate concentration of 4 mmol/L > *V*O_2max_. This ranking is based on the high correlations between 2000-m timetrial performance and power at *V*O_2max_ (*r* = 0.95, *p* < 0.001), power at 4 mmol/L (*r* = 0.92, *p* < 0.001), or *V*O_2max_ (*r* = 0.88, *p* < 0.001), respectively [38]. All outcomes were categorized as acute or chronic effects. In addition to these outcomes, relevant study information regarding author, year, number of participants, interventional data (weeks, frequency, duration per session, type of intervention), control condition, and PEDro scale scores were also recorded. Similar treatments are summarized in Table 2 for simplification of both networks. The corresponding interventions were classified as acute (≤ 7 days) or chronic (> 7 days).

**Table 2:** Overview of network treatments and number of studies using each treatment category are given in parentheses

Treatment	Description of treatments
Altitude training (4 studies)	Training (30–90 min, 3–4/wk), altitude training camp or sleeping under hypoxic/altitude conditions
β-alanine (4 studies)	β-alanine supplementation (0.8–6.4 g/day or 80 mg/kg/day); β-alanine is a non-essential amino acid synthesized in the liver and found in products of animal origin
Blood flow restriction training (1 study)	Rowing at low intensity with blood flow restricted legs (1 h/wk) in addition to usual endurance and resistance training
β-hydroxy β-methylbutyrate (1 study)	β-hydroxy β-methylbutyrate supplementation (3 g/kg/day); β-hydroxy-β-methylbutyrate is a metabolite derived from the essential amino acid leucine
β-hydroxy β-methylbutyrate and creatine (2 studies)	Combination of β-hydroxy β-methylbutyrate (3 g/kg/day) and creatine (0.04 g/kg/day) supplementation
Beetroot (2 studies)	Nitrate/beetroot supplementation (4.2–8.4 mmol/day); beetroot juice has a high inorganic nitrate (NO_3_^−^) content, a compound found naturally in vegetables and in processed meats, where it is used as a preservative
Black currant (1 study)	Black currant supplementation (750 mg/day), black currants are fruits/berries which are among plant products that are rich in flavonoids. Contains vitamin C, anthocyans, catechins, and querticin
Caffeine (6 studies)	Caffeine supplementation (3–9 mg/kg/day)
Cognitive fatigued (1 study)	Cognitive demanding task (Stroop task or arithmetic school test) prior to testing
Creatine (4 studies)	Creatine monohydrate supplementation (3–9 mg/kg/day)
Colostrum (1 study)	Bovine colostrum protein powder supplementation (60 g/day); bovine colostrum is the first milk secreted by cows after parturition and is a rich source of proteins, carbohydrates, fat, vitamins, minerals, and biologically active components such as antimicrobial molecules, immunoglobulins, and peptide growth factors
Elk velvet antler (1 study)	Elk velvet antler supplementation (560 mg/day)
Fasted state (1 study)	12 h with no food intake prior to testing
High-intensity training (8 studies)	Increased number of high-intensity training (above the second lactate threshold) sessions (about 2–3/wk)
Low-intensity training (3 studies)	Only low-intensity training (below first lactate threshold), in addition to usual resistance training
No resistance training (3 studies)	Only endurance training
Non-failure resistance training (2 studies)	Avoiding repetition failure during resistance training via predicted repetitions in reserve or velocity-based training, in addition to usual endurance training
Plyometric training (2 studies)	Plyometric jump training (2–3/wk), in addition to usual endurance and resistance training
Post-activation potentiation (2 studies)	5 × 5 s isometric or 2 × 10 s dynamic max efforts prior to testing; post-activation potentiation reverts to preconditioning exercise, which increases near-immediate muscular power and athlete’s performance
Precooling (1 study)	About 5–30 min’ cold exposure prior to testing
Preload (5 studies)	Several resistance training sessions, 25 s all-out arm crank intervals, 1-h low-intensity rowing (below first lactate threshold), or 6-min high-intensity rowing (above second lactate threshold) prior to testing
Prior weight reduction (5 studies)	About 4% weight reduction 24 h prior testing
Resistance rowing (1 study)	Rowing at low stroke rates, with high power per stroke (2–3/wk), in addition to usual endurance training
Resistance training only (1 study)	Only resistance training, with ≤ 30-min of endurance training per week
Strength endurance training (3 studies)	Resistance training with low load (< 70% 1RM) and high repetitions per sets (> 15), about 2–3/wk, in addition to usual endurance training
Sprint interval training (4 studies)	Short sprint interval sessions at supramaximal intensity (above P*V*O_2max_, 2.3/wk), in addition to usual endurance and resistance training
Spirulina (1 study)	Spirulina extract supplementation (1500 mg/day); spirulina, a microscopic and filamentous cyanobacterium, is considered a sustainable and eco-friendly microalga, playing an increasing role in alternative medicine
Shortened warm up (1 study)	Short warm-up duration or longer passive rest (about 30 min) prior to testing
Sodium bicarbonate (5 studies)	Sodium bicarbonate supplementation (0.3 g/kg/days; sodium bicarbonate (NaHCO_3_) is potentially effective in improving H^+^ buffering capacity
Sodium bicarbonate and caffeine (2 studies)	Combination of sodium bicarbonate (0.3 g/kg/day) and caffeine (3–6 mg/kg/day) supplementation
Sodium citrate (1 study)	Sodium citrate supplementation (0.5 g/kg/day); sodium citrate ingestion potentially increases the extracellular buffer capacity, augmenting the efflux of hydrogen ions (H^+^) and lactate from muscle cells to the extracellular fluid, therefore resulting in a less acidotic environment in muscle cells
Threshold training (4 studies)	Increased number of threshold sessions (between first and second lactate thresholds, about 2–3/wk)
Respiratory training (4 studies)	Breathing against resistance using respiratory training devices, in addition to usual endurance and resistance training
Respiratory preconditioning (2 studies)	Performing breathing exercises prior to testing
Usual preparation (31 studies)	Usual test preparation, including placebo (if nutritional supplementation was used for intervention)
Usual training (32 studies)	Usual rowing-specific endurance and resistance training, with about > 75% low-intensity training (below first lactate threshold), < 20% threshold training (between first and second lactate threshold), and < 10% high-intensity training (above second lactate threshold), including placebo (if nutritional supplementation was used for intervention)

Details of each included study are given in Table 3

### Statistical Analysis

The standardized mean difference (SMD) and 95% confidence intervals were calculated for all interventional treatments as a measure of treatment effectiveness. SMDs were calculated as differences between means divided by the pooled standard deviations (trivial: SMD < 0.2, small: 0.2 ≤ SMD < 0.5, moderate: 0.5 ≤ SMD < 0.8, large SMD ≥ 0.8) [40]. Subsequently, two separate network models were computed for acute and chronic effects. Therefore, a frequentist approach was chosen. To visualize the networks, a network graph was created for each network. The estimations of treatment effects were calculated based on a random-effects model [41].The control group was defined as usual preparation for the acute effects and usual training for chronic effects **and**  served as the reference treatment. A ranking was created based on the P-score of the individual treatments. The P-score represents the means of one-sided *P*-values under the normality assumption in a frequentist NMA [33]. This is interpreted as the mean extent of certainty that one intervention is superior to any other and is analogous to the surface under the cumulative ranking curve (SUCRA) [34] values of Bayesian NMA [33]. P-scores range from 0 to 100% with 0 and 1 being the theoretically worst and best treatment, respectively. Additionally, a forest plot was created to further visualize the ranking and effects of the treatments. Decomposed Q-statistics (within and between designs) were used to interpret potential heterogeneity and inconsistency. Heterogeneity and inconsistency were quantified using I^2^ [42]. Funnel plots were created to check for potential publication bias and Egger’s test for asymmetry of the funnel plot was used [43]. All calculations and presentational figures were made using the R software (version 4.1.1; The R Foundation for Statistical Computing) and the package ‘netmeta’ [44].

## Results

### Study Characteristics and Quality

**Fig. 1:**
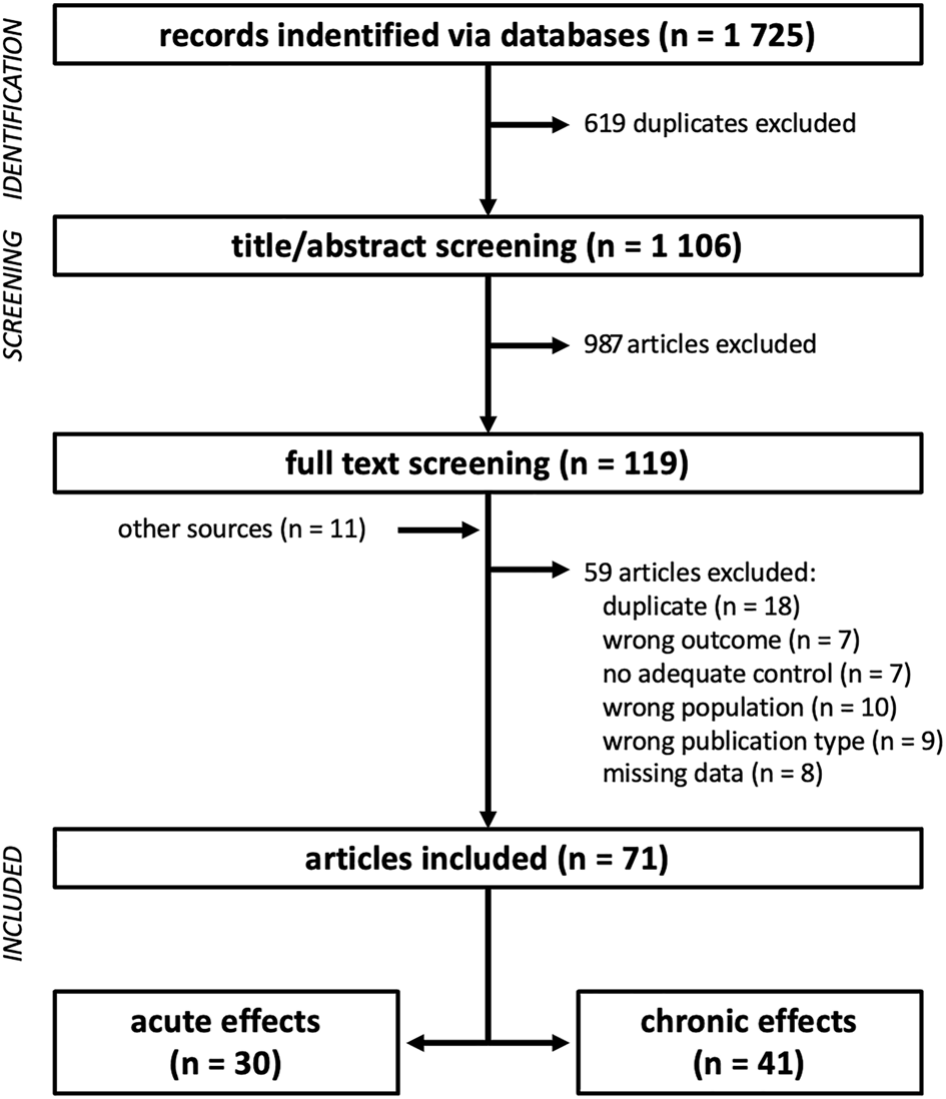
Flow chart of study screening and selection

After screening and study selection (Fig. 1), 71 studies were included in the NMA. The full list of selected studies, with the corresponding study details is displayed in Table 3. Overall, 1229 healthy rowers were examined, consisting of 237 female and 992 male rowers. Included trials enrolled on average 17.3 ± 8.7 participants per study (range 5–46) with an average age of 21.5 ± 3.0 years (range 11.0–30.4 years). The average study quality was high, (PEDro score; 8.5 ± 1.2; (range 6–10; Table 3). Apart from three four-armed study designs [30, 45, 46] and four three-armed study designs [22, 47–49], all studies employed a two-armed study design [7–18, 20, 21, 23–29, 31, 39, 50–65, 65–89].

*1RM* one-repetition maximum, *4minP* mean power of 4 min TT, *ET* endurance training, *HiT* high-intensity endurance training, *HR* heart rate, *HR*_*max*_ maximal heart rate, *ISO* isometric, *LiT* low-intensity endurance training, *NA* not available, *P4* power at 4 mmol/L lactate, *PAP* post-activation potentiation, *PPO* peak power output, *PVO*_*2max*_ power at VO_2max_, *RT* resistance training, *SiT* sprint interval training, *ThT* threshold-intensity endurance training, *TT* time trial, *TTE* time to exhaustion, *VO*_*2max*_ maximal oxygen consumption

### Acute and Chronic Effects Networks

**Fig. 2:**
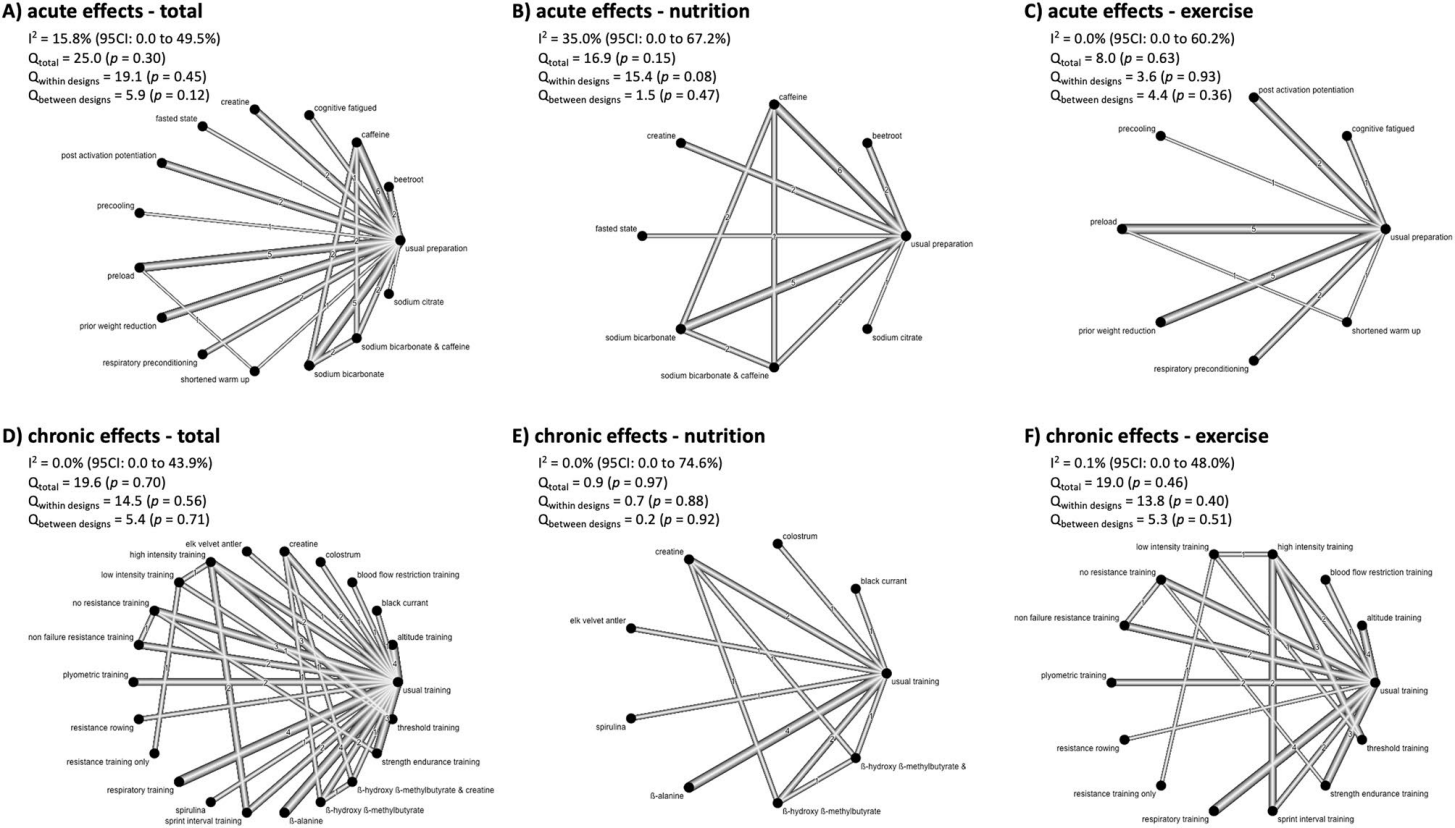
Network plots of the acute (**A**–**C**) and chronic (**D**–**F**) effects on rowing performance. Total (**A**, **D**), nutrition-related (**B**, **E**), and exercise-related (**C**, **F**) samples are displayed separately. In addition, *I*^2^, and Q statistic are given

In the acute-effects network (Fig. 2A), data from 30 studies (427 participants) representing 43 (pairwise comparison) effect sizes were included. The most common comparison was between caffeine vs. usual preparation (*n* = 6), followed by preload vs. usual preparation (*n* = 5), prior weight reduction vs. usual preparation (*n* = 5) and sodium bicarbonate vs. usual preparation (*n* = 5). The chronic-effects network (Fig. 2B) is based on 41 studies (822 participants) representing 50 (pairwise comparison) effect sizes. The most common comparisons were respiratory training vs. usual training (*n* = 4),  altitude training  vs. usual training (*n* = 4) and β-alanine versus usual vs. training (n = 43), followed by no resistance training  vs. training (*n* = 3), high intensity training  vs. threshold training (*n* = 3) and strength endurance training  vs. usual training (*n* = 3).

**Fig. 3:**
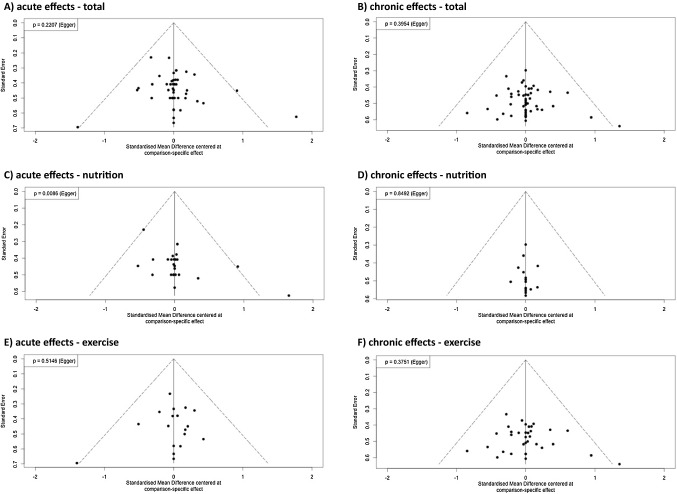
Funnel plots for the acute (**A**, **C**, **E**) and chronic effects on rowing performance (**B**, **D**, **F**) networks. Total (**A**, **B**), nutrition-related (**C**, **D**), and exercise-related (**E**, **F**) samples are displayed separately. In addition, Egger’s significances *(p* values*)* are given

Both the acute- and chronic-effects networks revealed low heterogeneity and non-significant heterogeneity (see *I*^2^ and Q statistics; Fig. 2), which applied to the total, nutrition-related, and exercise-related networks. In addition, funnel plot evaluations and non-significant Egger’s tests revealed no risk of bias for all networks (Fig. 3). Only the funnel plot of the chronic exercise network (Fig. 5D) revealed a significant Egger’s test result (*p* < 0.01). However, visual inspection indicated that this asymmetry was contrary to the corresponding publication bias.

**Fig. 4:**
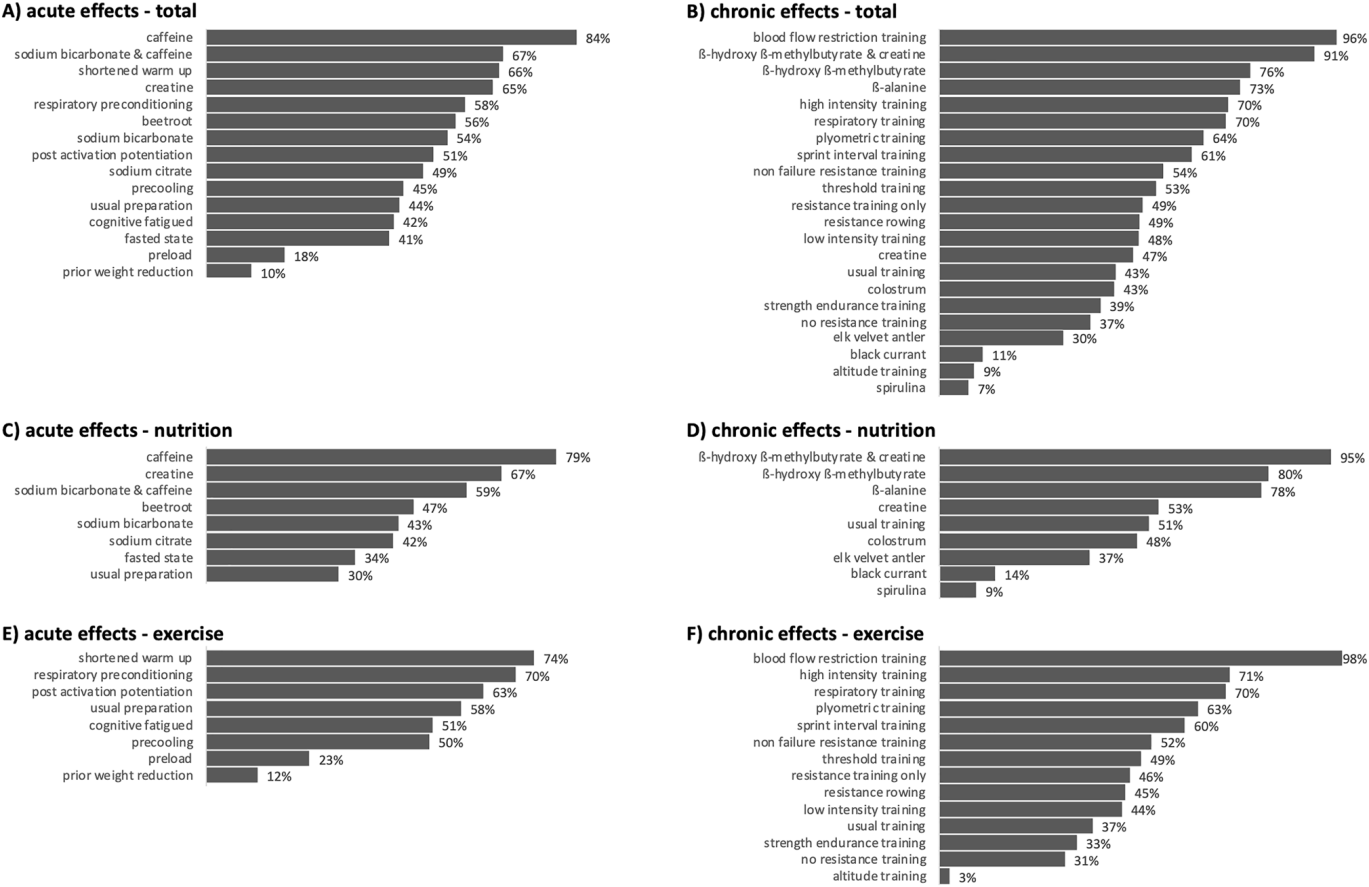
P-score rankings of the acute (**A**, **C**, **E**) and chronic (**B**, **D**, **F**) effects on rowing performance. Total (**A**, **B**), nutrition-related (**C**, **D**), and exercise-related (**E**, **F**) samples are displayed separately.

**Fig. 5:**
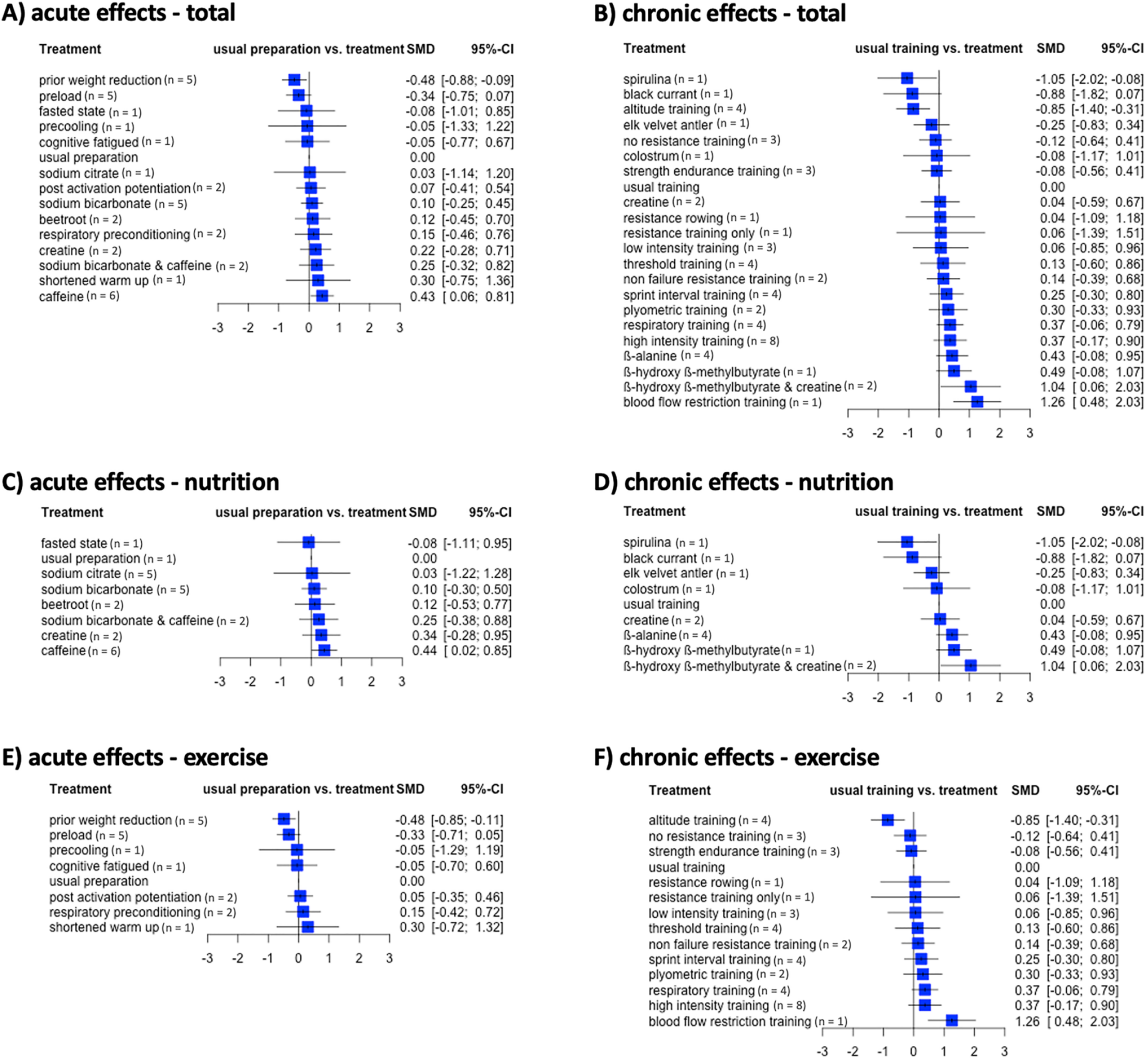
Forest plots for the acute (**A**, **C**, **E**) and chronic (B,D,F) effects on rowing performance networks. Total (**A**, **B**), nutrition-related (**C**, **D**), and exercise-related (**E**, **F**) samples are displayed separately. In addition, number of corresponding studies/comparisons are given in brackets.

Correspondingly, the P-score-based rankings of treatments are shown in Fig. 4. In addition, pairwise comparisons of both acute and chronic effects are presented as forest plots (Fig. 5). Thereby, nutrition-related and exercise-related data for both acute- and chronic-effects networks are displayed separately.

## Discussion

This is the first network meta-analytical review that reviewed acute and chronic effects of different nutritional supplementation strategies and exercise-based interventions on rowing performance. To the best of our knowledge, there has not been a summary or ranking of rowing-related interventions of this scale available to [March 7, 2022]. Our key findings indicated (i) favorable effects of caffeine supplementation on acute rowing time-trial performance, and (ii) large positive effects of blood flow restriction training and the combination of β-hydroxy-β-methylbutyrate and creatine supplementation on chronic adaptation of rowing performance indices. In contrast, our network analytical approach suggested small negative effects on acute rowing-related time-trial performance through prior weight reduction or extensive preload. Furthermore, chronic spirulina and black currant supplementations may hamper rowing performance improvements. Despite different acute and chronic interventional approaches on rowing performance, only a few rowing-specific meta-analyses on caffeine supplementation [19], preconditioning [32], and resistance training [7] are available. In contrast to the pairwise meta-analyses, our network analytical approach allowed not only two treatments to be compared, but many different treatments to be integrated into the network. Accordingly, the current study is the first to analyze acute and chronic effects of different nutritional supplementation strategies and exercise-based interventions on rowing performance.

**Table 3:** Description of included studies

Study	Sample	Age [y]	Duration	PEDro [a.u.]	Outcome	Interventions
Anderson et al. 2000 [50]	8 competitive rowers (0 males)	22.4 ± 3.0	Acute (≤ 1 wk)	9	2-km TT	1. Caffeine: 6–9 mg/kg caffeine 2. Usual preparation: placebo
Arend et al. 2021 [21]	10 high level rowers (10 males)	23.1 ± 3.8	Acute (≤ 1 wk)	8	TTE @90% P*V*O_2max_	1. Respiratory preconditioning: 8-min easy rowing @60–75% P*V*O_2max_; 2 × 30 inspirations @40% maximal inspiratory pressure using a POWERbreathe device; total time matched 2. Usual preparation: 8-min easy rowing @60–75% P*V*O_2max_; 5-min rest
Bond et al. 2012 [29]	14 well trained rowers (14 males)	16.7 ± 0.5	Acute (≤ 1 wk)	10	6 × 500 m TT	1. Beetroot: 5.5 mmol/days of NO_3_^−^ (nitrate), 6 days 2. Usual preparation: placebo
Bruce et al. 2000 [54]	8 well trained rowers (8 males)	> 18	Acute (≤ 1 wk)	10	2-km TT	1. Caffeine: 6–9 mg/kg caffeine 2. Usual preparation: placebo
Carr et al. 2011 [30]	8 well trained rowers (6 males)	> 18	Acute (≤ 1 wk)	9	2-km TT	1. Sodium bicarbonate and caffeine: 0.3 g/kg sodium bicarbonate; 6 mg/kg caffeine 2. Sodium bicarbonate: 0.3 g/kg sodium bicarbonate 3. Caffeine: 6 mg/kg caffeine 4. Usual preparation: placebo, cornflour
Carr et al. 2012 [55]	7 well trained rowers (4 males)	25.0 ± 11.7	Acute (≤ 1 wk)	9	2-km TT	1. Sodium bicarbonate: 0.3 g/kg sodium bicarbonate 1 × or 0.5 g/g sodium bicarbonate for 3 d 2. Usual preparation: placebo, ingested calcium carbonate
Christensen et al. 2014 [45]	12 elite rowers (11 males)	25.0 ± 2.0	Acute (≤ 1 wk)	10	2-min TT	1. Caffeine: 3 mg/kg caffeine 2. Sodium bicarbonate and caffeine: 3 mg/kg caffeine, 0.3 g/kg sodium bicarbonate 3. Usual preparation: placebo 4. Sodium bicarbonate: 0.3 g/kg sodium bicarbonate
Chwalbiñska-Moneta 2003 [56]	16 elite rowers (16 males)	23.9 ± 1.1	Acute (≤ 1 wk)	10	TTE	1. Creatine: creatine monohydrate 20 g/days, 5 days 2. Usual preparation: placebo, glucose 20 g/days, 5 days
Cornford and Metcalfe 2019 [57]	10 rowers (2 males)	21.0 ± 2.0	Acute (≤ 1 wk)	8	2-km TT	1. Fasted state: 12-h fasted prior testing 2. Usual preparation: usual preparation
Feros et al. 2012 [20]	10 elite rowers (9 males)	24.8 ± 2.6	Acute (≤ 1 wk)	8	1-km TT	1. Post-activation potentiation: isometric preconditioning contractions, 5 × 5 s/15 s 2. Usual preparation: time matched usual warm up
Filipas et al. 2018 [39]	18 rowers (11 males)	11.0 ± 1.1	Acute (≤ 1 wk)	10	1.5-km TT	1. Cognitive fatigued: cognitive demanding task (Stroop task or arithmetic school test) prior to testing 2. Usual preparation: placebo task (painting) prior to testing
Gee et al. 2011 [90]	8 rowers (8 males)	23.6 ± 6.8	Acute (≤ 1 wk)	8	2-km TT	1. Preload: heavy/exhaustion RT 24–48 h prior to testing 2. Usual preparation: normal preparation, no excessively exhausting training sessions 3–4 days before the test day
Gee et al. 2016 [67]	28 well trained rowers (28 males)	21.6 ± 4.0	Acute (≤ 1 wk)	8	2-km TT	1. Preload: 3 RT sessions during week before testing 2. Usual preparation: usual preparation, no excessively exhausting training sessions 3–4 days before the test day
Gharaat et al. 2020 [62]	9 elite rowers (9 males)	18.1 ± 1.1	Acute (≤ 1 wk)	10	2-km TT	1. Caffeine: 3–6 mg/kg caffeine 2. Usual preparation: placebo cellulose
Harat et al. 2020 [63]	40 well trained rowers (40 males)	20.0 ± 1.4	Acute (≤ 1 wk)	8	3-min TT	1. Post-activation potentiation: 5 × 5 s ISO PAP; dynamic PAP, 2 × 10 s max 2. Usual preparation: usual time matched warm up
Hobson et al. 2014 [65]	20 well trained rowers (20 males)	23.0 ± 4.0	Acute (≤ 1 wk)	10	2-km TT	1. Sodium bicarbonate: 0.3 g/kg sodium bicarbonate 2. Usual preparation: placebo
Hoon et al. 2014 [66]	10 highly trained rowers (10 males)	20.6 ± 2.5	Acute (≤ 1 wk)	9	2-km TT	1. Beetroot: 4.2–8.4 mmol NO_3_^−^ 2. Usual preparation: placebo
Martins et al. 2010 [31]	6 well trained rowers (6 males)	24.0 ± 6.0	Acute (≤ 1 wk)	10	2-km TT	1. Sodium citrate: 0.5 g/kg sodium citrate 2. Usual preparation: placebo
Mujika et al. 2012 [74]	14 highly trained rowers (14 males)	25.9 ± 5.3	Acute (≤ 1 wk)	8	2-km TT	1. Preload: 60-min warm up, with some sprints 2. Usual preparation: 30-min warm up, with some sprints
Penkman et al. 2008 [23]	7 well trained rowers (0 males)	22.4 ± 3.6	Acute (≤ 1 wk)	8	2-km TT	1. Prior weight reduction: 3.5% body mass reduction 24 h prior, using restricting fluid intake and no food consumption 2. Usual preparation: normal preparation
Purge et al. 2017 [77]	9 well trained rowers (9 males)	24.6 ± 7.1	Acute (≤ 1 wk)	8	2-km TT	1. Preload: usual warm up + 25" all-out arm crank pre-load 2. Usual preparation: usual warm up; no excessively exhausting training sessions 3–4 days before the test day
Rossiter et al. 1996 [79]	38 well trained rowers (28 males)	22.6 ± 4.4	Acute (≤ 1 wk)	9	1-km TT	1. Creatine: creatine 0.25 g/kg/days, 5 days 2. Usual preparation: placebo
Skinner et al. 2010 [81]	10 well trained rowers (10 males)	20.6 ± 1.4	Acute (≤ 1 wk)	10	2-km TT	1. Caffeine: 2–6 mg/kg caffeine 2. Usual preparation: placebo
Slater et al. 2005 [83]	17 well trained rowers (8 males)	22.5 ± 4	Acute (≤ 1 wk)	7	1.8-km TT	1. Prior weight reduction: about 4% body mass reduction 24 h prior 2. Usual preparation: normal preparation
Slater et al. 2006a [84]	17 well trained rowers (8 males)	22.5 ± 4	Acute (≤ 1 wk)	7	2-km TT	1. Prior weight reduction: about 4% body mass reduction 24 h prior 2. Usual preparation: normal preparation
Slater et al. 2006b [82]	16 well trained rowers (16 males)	20.7 ± 2.2	Acute (≤ 1 wk)	7	2-km TT	1. Prior weight reduction: about 4% body mass reduction 24 h prior 2. Usual preparation: normal preparation
Slater et al. 2007 [85]	12 well trained rowers (12 males)	19.6 ± 1.6	Acute (≤ 1 wk)	9	2-km TT	1. Prior weight reduction: 3.5% body mass reduction 24 h prior, using restricting fluid intake and no food consumption 2. Usual preparation: normal preparation
Sousa et al. 2014 [49]	6 highly trained rowers (6 males)	22.9 ± 4.5	Acute (≤ 1 wk)	8	TTE @P*V*O_2max_	1. Preload: 2 min @20% PPO, 6 min > lactate threshold, 7 min rest 2. Usual preparation: usual warm up, 2 min @20% PPO, 6 min below lactate threshold, 7 min rest 3. Shortened warm up: 2 min @20% PPO, 7 min rest
Spitz et al. 2014 [22]	5 well trained rowers (3 males)	23.4 ± 1.6	Acute (≤ 1 wk)	8	2-km TT	1. Precooling: 5 or 30 min cold exposure prior to TT 2. Usual preparation: normal preparation
Volianitis et al. 2001a [88]	14 well trained rowers (7 males)	20.0 ± 0.8	Acute (≤ 1 wk)	7	6-min TT	1.Respiratory preconditioning: usual warm up, with respiratory warm up using the POWERbreathe device 2. Usual preparation: usual warm up
Akca and Aras 2015 [14]	20 competitive rowers (20 males)	21.8 ± 2.4	Chronic (4 wk)	8	2-km TT	1. High intensity training: HiT, 2/wk, 8 × 2.5 min @90% 2kP/3 min @40% 2kP 2. Sprint interval training: SiT, 2/wk, 10 × 30 s @150% 2kP/4 min @40% 2kP; total time matched
Baguet et al. 2010 [51]	18 elite rowers (17 males)	22.9 ± 4.2	Chronic (7 wk)	10	2-km TT	1. β-alanine: β-alanine, 5 g/days, in addition to RT and ET 2. Usual training: placebo/maltodextrin, 5 g/days, in addition to RT and ET
Beasley et al. 2018 [52]	27 rowers (27 males)	24.0 ± 5.0	Chronic (4 wk)	10	30-min TT	1. β-alanine: 2.4–4.8 g/days, in addition to RT and ET 2. Usual training: cornflour placebo 4.8 g/days, in addition to usual RT and ET
Brinkworth et al. 2002 [53]	13 elite rowers (13 males)	20.6 ± 3.2	Chronic (9 wk)	10	4-min TT	1. Colostrum: 60 g/bovine colostrum protein powder 2. Usual training: placebo
Chinapong et al. 2021 [17]	14 rowers (14 males)	20.0 ± 1.7	Chronic (6 wk)	8	*V*O_2max_	1. Altitude training: 30-min row in normobaric hypoxic chamber (3000 m), 4/wk, in addition to usual ET and RT 2. Usual training: usual time matched ET and RT
Driller et al. 2009 [15]	10 well trained rowers (5 males)	19.0 ± 2.0	Chronic (4 wk)	8	2-km TT	1. High intensity training: HiT, 2/wk, 8 × 2.5 min @90% PPO from incremental exercise test/40% PPO till HR < 70% HR_max_ 2. Threshold training: ThT, 30–45´ @2–3 mmol/L
Ducker et al. 2013 [59]	16 elite rowers (16 males)	26.0 ± 9.0	Chronic (4 wk)	10	2-km TT	1. β-alanine: 80 mg/days/kg, β-alanine 2. Usual training: placebo
Durkalec-Michalski and Jeszka 2015 [60]	16 elite rowers (16 males)	19.5 ± 1.4	Chronic (12 wk)	10	*V*O_2max_	1. β-hydroxy β-methylbutyrate: 3 g/days, HMB ( β-hydroxy- β-methylbutyric acid 2. Usual training: placebo, 3 g/days, maltodextrin
Ebben et al. 2004 [61]	26 rowers (0 males)	20.0 ± 1.0	Chronic (8 wk)	8	2-km TT	1. Strength endurance training: low load, high reps RT, 15–32 reps/sets, 2/wk, in addition to usual ET 2. Usual training: high load, low reps, 5–12 reps/set, 2/wk, in addition to usual ET
Egan-Shuttler et al. 2017 [8]	16 well trained rowers (16 males)	16.0 ± 0.7	Chronic (4 wk)	7	500-m TT	1. Plyometric training: 30 min plyometric exercise, 3/wk, in addition to usual RT and ET 2. Usual training: 30 min steady-state cycling @ventilatory threshold, in addition to usual RT and ET
Fernández-Landa et al. 2020 [46]	28 elite rowers (28 males)	30.4 ± 4.7	Chronic (10 wk)	10	P4	1. Creatine: creatine monohydrate 0.04 g/kg/days 2. Usual training: placebo 3. β-hydroxy β-methylbutyrate and creatine: creatine monohydrate 0.04 g/kg/days; β-hydroxy-β-methylbutyrate 3 g/days 4. β-hydroxy β-methylbutyrate: 0.04 g/kg/days; β-hydroxy-β-methylbutyrate 3 g/days
Forbes et al. 2011 [10]	21 rowers (9 males)	22.4 ± 9.9	Chronic (10 wk)	10	2-km TT	1. Respiratory training: respiratory muscle training, 1–2/wk using PowerLung device, in addition to usual RT and ET 2. Usual training: placebo respiratory training, in addition to usual RT and ET
Gallagher et al. 2010 [47]	18 rowers (18 males)	20.5 ± 0.9	Chronic (8 wk)	8	2-km TT	1. Usual training: high load low reps resistance training; 2/wk; 3–5 sets, 1–5 reps, in addition to usual ET 2. Strength endurance training: low load high reps resistance training; 2/wk; 2–3 sets, 15–30 reps, in addition to usual ET 3. No resistance training: only ET; 2 sessions less than other groups
Held et al. 2020 [16]	31 well trained rowers (23 males)	21.8 ± 3.4	Chronic (5 wk)	8	P*V*O_2max_	1. Blood flow restriction training: BFR 2 × 10 min @LIT, 3/wk, in addition to usual RT and ET 2. Usual training: usual RT and ET
Held et al. 2021 [64]	21 well trained rowers (17 males)	19.6 ± 2.1	Chronic (8 wk)	8	P*V*O_2max_	1. Non-failure resistance training: RT with max. 10% velocity loss, 2/wk, in addition to usual ET 2. Usual training: usual RT and ET, including traditional RT to repetition failure
Hinckson et al. 2006 [18]	12 elite rowers (3 males)	21.8 ± 3	Chronic (3 wk)	10	5-km TT	1. Altitude training: 90 min altitude while resting, using an inhaler device, 3/wk, in addition to usual RT and ET 2. Usual training: placebo inhaler, 90 min, 3/wk, in addition to usual RT and ET
Hobson et al. 2013 [24]	20 well trained rowers (20 males)	23.0 ± 4.0	Chronic (4 wk)	10	2-km TT	1. β-alanine: 6.4 g/days β-alanine, in addition to usual RT and ET 2. Usual training: placebo
Ingham et al. 2008 [68]	18 highly trained rowers (18 males)	24.0 ± 4.2	Chronic (12 wk)	8	2-km TT	1. Threshold-training: 70% LiT, 30% ThT/HiT 2. Low intensity-training: 100% LiT
Izquierdo-Gabarren et al. 2009 [48]	37 rowers (37 males)	26.3 ± 5.4	Chronic (8 wk)	8	20-min TT	1. Non-failure resistance training: non-repetition failure-based RT, 2/wk, in addition to usual ET 2. Usual training: usual repetition failure-based RT, in addition to usual ET 3. No resistance training: no RT, only usual ET
Jaakson and Mäestu 2012 [69]	12 elite rowers (12 males)	21.2 ± 1.8	Chronic (4 wk)	7	TTE @95% PPO	1. Resistance rowing: using rowing with high pressure as RT, 2/wk, in addition to usual ET 2. Usual training: traditional RT, 2/wk, in addition to usual ET
Jensen et al. 1993 [91]	18 elite rowers (18 males)	22.5	Chronic (3 wk)	6	*V*O_2max_	1. Altitude training: altitude training camp at 1822 m 2. Usual training: sea-level training camp
Juszkiewicz et al. 2018 [25]	19 elite rowers (19 males)	20.2 ± 0.8	Chronic (6 wk)	10	2-km TT	1. Spirulina: 1500 mg/days spirulina extract 2. Usual training: placebo
Kirchenberger et al. 2021 [70]	17 highly trained rowers (17 males)	15.3 ± 1.3	Chronic (8 wk)	8	2-km TT	1. High-intensity training: 4 × 2´ @95% HR_max_/1 min rest, 2/wk, in addition to usual RT and LiT 2. Low-intensity training: only RT and LiT
Koparal et al. 2021 [71]	24 rowers (24 males)	20.2 ± 1.2	Chronic (8 wk)	8	2-km TT	1. High-intensity training: combination of HiT (30–90 s intervals @75–90% 2kP) and RT, in addition to usual ET 2. Usual training: usual ET and RT
Kramer et al. 1993 [9]	28 rowers (28 males)	20.6 ± 1.7	Chronic (9 wk)	8	2.5-km TT	1. Plyometric training: plyometric exercises, 3/wk, in addition to usual RT and ET 2. Usual training: usual ET and RT
Lawton et al. 2012 [72]	22 elite rowers (12 males)	24.4 ± 3.9	Chronic (8 wk)	7	P4	1. Usual training: RT, 2–4/wk, in addition to usual ET 2. No resistance training: no RT, time-matched ET
Liu et al. 2003 [73]	6 elite rowers (6 males)	19.0 ± 3.0	Chronic (3 wk)	7	P4	1. Resistance training only: RT @55–75% 1RM, 6/wk, mean lactate of 7.1 ± 2.2 mmol/L, 30 min rowing/wk 2. Low-intensity training: LiT only, about 90 min/days, @1.47 ± 0.42 mmol/L, 10 min RT/wk
Neykov et al. 2019 [75]	16 elite rowers (16 males)	17.1 ± 0.8	Chronic (4 wk)	7	P4	1. Altitude training: sleep with altitude mask connected to hypoxicators (1600–2800 m), in addition to usual ET and RT 2. Usual training: usual RT and ET
Ní Chéilleachair et al. 2017 [76]	19 well trained rowers (14 males)	22.0 ± 4.0	Chronic (8 wk)	8	2-km TT	1. High-intensity training: LiT with HiT 2/wk, ThT 2/wk 2. Threshold-training: LiT with ThT 2/wk
Richer et al. 2016 [12]	16 well trained rowers (10 males)	22.0 ± 3.0	Chronic (8 wk)	8	2-km TT	1. Sprint interval training: usual ET and RT, with 6 SiT sessions 2. Usual training: usual ET and RT
Riganas et al. 2008 [78]	19 elite rowers (12 males)	20.9 ± 3.8	Chronic (6 wk)	7	2-km TT	1. Respiratory training: inspiratory muscle training, 30 min, 5/wk, in addition to usual RT and ET 2. Usual training: usual ET and RT
Riganas et al. 2019 [11]	36 well trained rowers (20 males)	19.4 ± 7.4	Chronic (6 wk)	8	2-km TT	1. Respiratory training: inspiratory muscle training, 30 min, 5/wk, using POWERbreathe device, in addition to usual ET and RT 2. Usual training: usual ET and RT
Shing et al. 2013 [80]	7 well trained rowers (5 males)	19.0 ± 1.2	Chronic (4 wk)	10	4-min TT	1. High-intensity training: HiT 8 × 2.5´ @90% 4minP /4´ @40% 4minP till < 70% HR_max_, 2/wk, in addition to usual ET and RT 2. Threshold-training: 35–40 min @2–3 mmol/L, 2/wk, in addition to usual RT and ET
Skarpańska-Stejnborn et al. 2006 [26]	19 elite rowers (19 males)	20.5 ± 1.2	Chronic (6 wk)	10	2-km TT	1. Black currant: black currant 250 mg, 3/days 2. Usual training: placebo
Stevens et al. 2015 [86]	16 well trained rowers (16 males)	20.0 ± 1.7	Chronic (4 wk)	7	2-km TT	1. Sprint interval training: 4–6 × 1 min all out /2.5–4 min rest, 2/wk, in addition to usual ET and RT 2. Usual training: time matched usual ET and RT
Syrotuik et al. 2005 [27]	46 well trained rowers (25 males)	25.3 ± 5.3	Chronic (10 wk)	10	2-km TT	1. Elk velvet antler: 280 mg, 2/days 2. Usual training: placebo
Syrotuik et al. 2001 [28]	22 rowers (12 males)	23	Chronic (6 wk)	10	2-km TT	1. Creatine: creatine monohydrate, 5 d load with 0.3 g/kg/days; then 0.03 g/kg/days 2. Usual training: placebo
Thiele et al. 2020 [92]	26 elite rowers (0 males)	13.2 ± 0.5	Chronic (9 wk)	7	700-m TT	1. Usual training: heavy load, low reps, RT, 75–95% 1RM, 12 reps, 2/4, 1. volume matched, in addition to usual ET 2. Strength endurance training: low load, high reps, RT, 2/wk, 50–60% 1RM, in addition to usual ET
Treff et al. 2017 [87]	14 elite rowers (14 males)	20.0 ± 1.5	Chronic (11 wk)	7	2-km TT	1. High-intensity training: polarized training, more HiT, less ThT 2. Usual training: usual pyramidal training
Turner et al. 2021 [13]	24 elite rowers (17 males)	21.7 ± 3.1	Chronic (3 wk)	8	2-km TT	1. Sprint interval training: 8 SiT sessions, 3 × (7 × 30 s all out/1 min rest), in addition to usual ET and RT 2. High-intensity training: 8 HiT sessions, 8 × 2.5 min /2.5 min rest, in addition to usual ET and RT
Volianitis et al. 2001b [89]	14 well trained rowers (14 males)	23.8 ± 3.8	Chronic (11 wk)	10	6-min TT	1. Respiratory training: respiratory training, using POWERbreathe device, 5 min, 2/days 2. Usual training: placebo training, in addition to usual RT and ET

### Acute Effects

Acute caffeine supplementation scored the highest in the P-score ranking, with small- to moderate- positive effects. In line with these findings, previous multisports-based meta-analytical reviews revealed relevant improvements in time trial performance  via acute creatine supplementation [93, 94]. Similarly, a rowing-related meta-analysis [19] revealed acute timetrial performance enhancement effects via caffeine supplementation, which is in line with our findings. Although a systematic review [95] and a meta-analysis [96] showed acute multisports-based timetrial performance enhancements via beetroot supplementation, our network-analytical approach revealed only trivial effects on rowing time-trial performance. Similarly, several meta-analytical reviews have revealed improved muscular endurance [97], 200–400 m swimming performance [98], and (running or cycling) time to exhaustion performance [99] via acute sodium bicarbonate supplementation, whereas our findings revealed only trivial effects. These contrasting findings may be due to the small number of rowing-related studies on beetroot (*n* = 2) and sodium bicarbonate (*n* = 1) supplementation in our network model. Furthermore, our network revealed only trivial effects of acute creatine supplementation on the 2000-m time-trial performance. These findings are in line with those of previous multisports-based meta-analyses, since acute creatine supplementation increased only time-trial performance ≤ 3 min [100] and has even shown negative effects on *V*O_2max_ [101]. Based on the P-score ranking, our network showed that the effect of sodium bicarbonate on performance was enhanced by  its combination with caffeine. In contrast, the effects of caffeine appeared to be impaired when combined with sodium bicarbonate. However, because of considerable overlap in the effect sizes (95% confidence intervals of standard mean differences), these differences are difficult to interpret. Future studies should investigate the effects of combining various supplementation strategies. Apart from nutritional supplementation strategies, our acute network revealed merely trivial effects of precooling on the 2000-m time-trial performance (under usual temperature conditions ≤ 23 °C). These findings were in line with previous multisports-based meta-analyses, which revealed enhancement effects of precooling on time-trial performance only in hot environments [102, 103]. Likewise, a multisports-based meta-analytical review revealed small performance-enhancing effects on jumping, throwing, and sprint performance via post-activation potentiation (PAP) approaches [104]. In contrast, our data revealed that these PAP effects are only trivial for rowing-related 2000-m time-trial performance improvements. In addition, only one meta-analytical review indicated that an adequate warm-up procedure could improve performance [105]. Nevertheless, our network analytical approach indicated small but relevant negative effects of prior weight reduction and preload (heavy resistance training, high-intensity training or longer low-intensity training prior to testing) on subsequent rowing-specific time-trial performances. Therefore, weight reduction, heavy resistance training, high-intensity training, and longer low-intensity training should be strictly avoided within the 48 h prior to a crucial time-trial testing.

### Chronic Effects

Our network analytical approach revealed large (combination of β-hydroxy-β-methylbutyrate and creatine), small (β-hydroxy-β-methylbutyrate or β-alanine), and trivial (creatine, colostrum, or elk velvet antler) beneficial effects of chronic nutritional supplementation strategies on the 2000-m timetrial performance. Interestingly, the combination of β-hydroxy-β-methylbutyrate and creatine induced more pronounced beneficial effects on rowing timetrial performance than the separate supplementation of β-hydroxy-β-methylbutyrate or creatine. The positive effects of β-hydroxy-β-methylbutyrate and creatine were partly surprising, since previous multisports-based meta-analyses and systematic reviews revealed (i) only performance-enhancing effects via creatine supplementation when timetrial duration was ≤ 3 min [100]; (ii) negative effect of creatine supplementation on maximal oxygen uptake [101]; and (iii) no effects on hypertrophy or strength if β-hydroxy-β-methylbutyrate was combined with resistance training [106]. Apart from this, other multisports-based meta-analyses [107, 108] revealed only small beneficial effects of chronic β-alanine supplementation on endurance performance indices, which was confirmed by our findings. Another recent meta-analysis revealed positive effects of spirulina supplementation on oxidative stress and pro-inflammatory biomarkers [109], systolic and diastolic blood pressure [110], and body weight reduction in obese individuals [111]. However, our network analytical approach revealed that these positive effects of spirulina supplementation are not transferable to improved timetrial rowing performance. In fact, based on the P-score ranking and calculated effect sizes, negative effects on rowing-specific performance might be expected. Similarly, our results show trivial to large negative effects of black currant supplementation, although a previous multisports-based meta-analysis showed only a small, but relevant, positive effect on sport performance, with no known detrimental side effects [112]. These contrasting findings may be explained by different intervention durations. While black currant is usually supplemented for only about seven days [112], the rowing study, which is integrated in the current network analytical approach, lasted six weeks [26]. Therefore, future research on black currant should target different intervention durations. Furthermore, these partly contrasting findings may be due to the fact that only one spirulina and one black currant supplementation study was included in our network analytical approach.

Apart from these supplementation strategies, numerous previously published meta-analyses have demonstrated the beneficial effects of low-intensity and threshold-intensity training [113], high-intensity training [113, 114], and sprint-interval training [114, 115] on relevant endurance performance surrogate parameters such as *V*O_2max_, lactate threshold power, or timetrial performance. Thereby, our network also corroborated positive but trivial effects of threshold-training, high-intensity training, and sprint-interval training on rowing-specific timetrial performance. These varying magnitudes of the effect sizes could be due to the comparative conditions used in each case: Whereas pairwise meta-analyses selected a comparison condition that was substantially contrasting (e.g., low- vs high-intensity training) [113–115], we chose the usual rowing training as the reference intervention for our network. Since usual rowing training also contains a certain amount of threshold-, high-intensity, and sprint-interval training, the effects could partially overlap, which might account for the lower effect sizes. This usual training comparator was chosen because it best represented the actual training of successful rowers.

Based on the P-score ranking and the calculated effect sizes, our network indicated that respiratory training via breathing against resistance has similar to higher effects on rowing-specific performance than threshold-, high-intensity, and sprint-interval training. Similarly, the positive effects of respiratory training on sports performance were concluded in a multisports-based meta-analysis [116]. These authors assumed that a more inclined progression of respiratory training intensity may induce even greater performance improvement [116]. Regarding resistance training, several multisports-based meta-analytical [117] and systematic reviews [118] concluded that the implementation of resistance training in addition to traditional sport-specific training improves endurance performance, mainly through improvements in the energy cost of locomotion, maximal power, and maximal strength. A recent rowing-related systematic review and meta-analysis [7] indicated that resistance training is effective in improving lower limb maximal strength and sport-specific performance in rowers. While this rower-specific meta-analysis was based on nine studies, our network analytical approach was able to consider a total of 41 chronic intervention studies which were linked by combining direct and indirect evidence. Overall, the positive effects of resistance training are also reflected by our results. However, the different resistance training approaches show only trivial effects on the rowing-specific timetrial performance which are similar to non-resistance training approaches. These multifaceted approaches are also reflected in high-performance rowing, as successful rowing carriers can be achieved both with and without resistance training [119]. Interestingly, non-failure-based resistance training approaches such as velocity-based training [64] or repetition in reserve-based training [48] scored similar or even slightly better than the other resistance training approaches in our network. These findings are supported by a recent multisports-based meta-analysis [120] that concluded that resistance training to muscle failure does not seem to be required for gains in strength and muscle size [120]. Overall, several researchers concluded that moderate strength training volume and training not to repetition failure may be more favorable for achieving greater strength gains, muscle power, and rowing performance than with higher training volumes to repetition failure [48, 121, 122].

Previous non-rowing–related meta-analyses have revealed, besides improved vertical jump [123] and repeated sprint [124] abilities, endurance running performance improvements [125]. These improvements have been mainly attributed to plyometric exercises. Likewise, our network analytical approach revealed positive effects on rowing performance via plyometric training. However, the two included studies showed partly contradictory results. While one intervention study (*n* = 18, 4 weeks) revealed rowing-specific performance improvements through plyometric training [8], another intervention study (n=24, 9 weeks) observed no rowing-specific performance improvements [9]. These contradictory findings may be partly explained by methodological issues. For example, the sequence of stretching and contraction of a muscle tendon unit is described as a stretch–shortening cycle (SSC) [126]. In addition, an SSC enables up to 50% higher muscle force, work, and power output during the shortening phase of the SSC compared to isolated muscle shortening [127–129]. Considering that usual rowing results in a notable performance enhancement of ~ 10% compared to purely concentric rowing [130], it has been speculated that this is due to SSC-based mechanisms at the muscle level [130–132]. A differentiation between slow (> 250 ms) and fast SSC (< 250 ms) must be considered in discipline-specific movement analyses and training [133, 134]. Furthermore, training adaptations in the fast SSC are not necessarily transferable to performance increases in the slow SSC (and vice versa) [126, 134–136]. For rowing, it has been recently shown that examinations of surface electromyographic activity of selected leg muscles (*m. vastus medialis* and *m. gastrocnemius medialis*) showed no pre-activation or reflex activity, which implies that any form of muscle action in the fast SSC domain does not reflect discipline-specific muscle actions and could hamper rowing performance enhancement during training and competitions [132]. These SSC mechanism are rescently confirmed on the fascicle level in rowing. Since both rowing-related plyometric intervention studies [8, 9] used slow and fast SSC exercises to different extents, a comparison of the results is difficult. Accordingly, further research on the effects of plyometric training in rowers with application of exclusively slow SSC exercises is needed.

Although previous multisports-based meta-analyses [137, 138] have revealed improved endurance adaptations via altitude or hypoxic training, our network suggested that even performance declines via altitude training compared to usual rowing training. As the effect of altitude training is highly dependent on the protocol employed (e.g., sleep high, train low vs train high, sleep low) [139] and the limited number of included studies (*n* = 3), future rowing-related research should challenge or confirm this finding.

In contrast, our P-score ranking and calculated effect sizes showed superior adaptation via blood flow restriction training. Although these results were based on only one included study [16], they were confirmed by numerous multisports-based meta-analyses [140–142]. Thereby, numerous positive effects of blood flow restriction training such as increased strength, hypertrophy, and endurance adaptations have been reported [140–142].

### Limitations

One limitation is that the findings on individual treatments are in some cases based on only a small amount of direct evidence (Table 2). However, the heterogeneity and consistency of the data showed that the resulting network is valid in each case. Regardless, current data do not examine sex-specific differences due to gender issues in bioavailability. As only 20% (*n* = 237) of the included participants were female, the results should be cautiously generalized for female athletes. From a total of 71 included studies, 21 studies used elite rowers [18, 20, 25, 45, 46, 51, 53, 56, 60, 62, 69, 72, 73, 91]. These studies examined supplementation strategies like caffeine [45, 62], sodium bicarbonate with caffeine [45], creatine [46, 56], β-alanine [51, 59], colostrum [53], β-hydroxy β-methylbutyrate [60], β-hydroxy β-methylbutyrate with creatine [46], and spirulina [25]. Furthermore, post-activation potentiation [20], altitude training [18, 91], resistance rowing [69], non-resistance training [72], and resistance training only [73] were also examined in these studies with elite rowers. However, it was not imposssible to integrate a non-elite comparison into the respective networks. The current network analytic approach also integrated non-elite rower studies on caffeine, creatine, post-activation potentiation, β-alanine, colostrum, altitude training, and non-resistance training. In contrast, for sodium bicarbonate with caffeine, β-hydroxy β-methylbutyrate, β-hydroxy β-methylbutyrate with caffeine, resistance rowing and resistance training only, Therefore, based on the current network analytical data, it cannot be determined whether the findings regarding sodium bicarbonate with caffeine [45], β-hydroxy β-methylbutyrate [60], β-hydroxy β-methylbutyrate with caffeine [46], resistance rowing [69], and resistance training only [73] are also valid for less trained rowers.

The strengths of this study outweigh potential limitations of this network meta-analysis. These strengths include (i) the large number of included studies and overall comparisons, (ii) the robust homogeneity and consistency of the formed networks, and (iii) the methodological quality of the included studies (PEDro scores > 6). Additionally, most of the findings in this analysis are a solid condensation of many trials and are largely consistent with previous literature, further supporting the plausibility of these findings. With all this in mind, it is reasonable to assume that this network meta-analysis provides valuable and important evidence despite its limitations. In addition, the current study enabled the first meta-analytical investigation of rowing-specific findings on plyometric training [8, 9], respiratory training [10, 11], sprint-interval training [12, 13], high-intensity training [14, 15], blood flow restriction methods [16], altitude training [17, 18], weight loss management [23], β-alanine [24], spirulina [25], black currant [26], elk velvet antler [27], creatine monohydrate [28], beetroot [29], sodium bicarbonate [30], and sodium citrate [31].

## Conclusion

This network meta-analytical review revealed (i) moderate positive effects of caffeine supplementation on acute rowing timetrial performance; (ii) small to moderate negative effects on acute rowing-related time-trial performance via prior weight reduction or extensive preload; (iii) large positive effects of blood flow restriction training and the combination of β-hydroxy-β-methylbutyrate and creatine supplementation on (chronic) improvement of rowing performance indices, and (iv) large impairment effects of rowing performance adaptations via chronic spirulina and black currant supplementation. Overall, these findings indicate that the choice of the nutritional supplementation strategy and the exercise training approach has a meaningful impact on the magnitude of the effects and should therefore be carefully considered. Future research should focus on the optimal combination of nutritional and exercise modalities.

## Declarations

### Data availability

The datasets generated during and/or analyzed during the current study are available from the corresponding author on reasonable request.
